# A Case of Adult-Onset Still's Disease with Positive Antinuclear Antibodies

**DOI:** 10.7759/cureus.10761

**Published:** 2020-10-01

**Authors:** Harini Lakshman, Pal Satyajit Singh Athwal, Anitha Gondi, Sandaljit Dhillon, Basim A Towfiq

**Affiliations:** 1 Internal Medicine, Michigan State University at Hurley Medical Center, Flint, USA; 2 Internal Medicine, Saraswathi Institute of Medical Sciences, Hapur, IND

**Keywords:** adult-onset still’s disease, antinuclear antibodies

## Abstract

Adult-onset Still's disease (AOSD) is a rare inflammatory disorder involving multiple systems. It can present a wide range of symptoms like maculopapular rash, fever, and arthralgia, which may overlap with many other disorders, making it difficult to diagnose. Unknown etiology and no diagnostic tests further make it complex to establish the diagnosis of AOSD. We report the case of a 30-year-old female who presented with persistent rash, joint pain, and fever, along with positive antinuclear antibodies (ANA), diagnosed with this condition. The patient improved with corticosteroids and the plan is to start disease-modifying antirheumatic drugs (DMARDs) after tapering off steroids.

## Introduction

In 1897, George Frederic Still first described juvenile idiopathic arthritis (JIA) in 22 children, followed by Bywaters who later on defined adult-onset Still's disease (AOSD) [1-2]. AOSD is a rare autoimmune condition with unknown etiology. Clinically, it presents as a maculopapular rash, fever, arthralgia, leukocytosis, and raised acute phase reactants. It is a diagnosis of exclusion, and differentials include a wide range of infectious, autoimmune, neoplastic pathologies. Yamaguchi’s criteria can be used to diagnose after the exclusion of other disorders [3]. There is no diagnostic lab test though serum ferritin can be used for monitoring and consideration for AOSD [4]. Non-steroidal anti-inflammatory drugs (NSAIDs), corticosteroids, and rheumatological agents are the mainstay of treatment [5]. We report a case of AOSD diagnosed on the basis of the Yamaguchi criterion and weakly positive antinuclear antibodies (ANA).

## Case presentation

A 30-year-old female with a past medical history significant for protein C deficiency and a history of pulmonary embolism comes with an urticarial rash associated with arthralgia. She did not have any history of fever or joint pain at that time. The episode started recently and was associated with high fever and significant joint pain and stiffness. She was seen in urgent care and treated with prednisone 20 mg. Her rash remained stable but she still had swelling and tenderness of bilateral knee and ankle joints and bilateral wrist and elbow joints.

**Figure 1:**
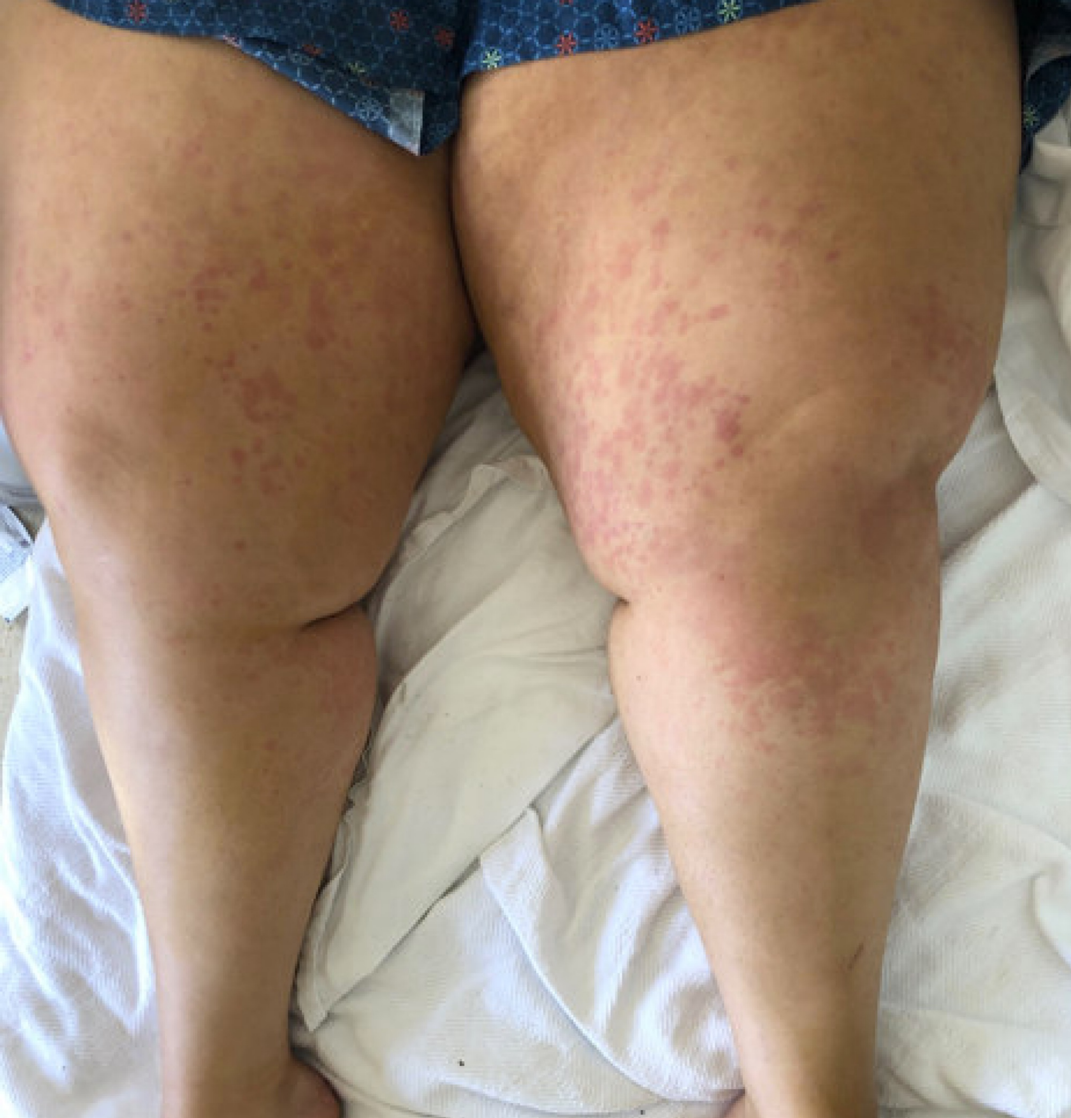
Rash distributed over both lower limbs

**Figure 2:**
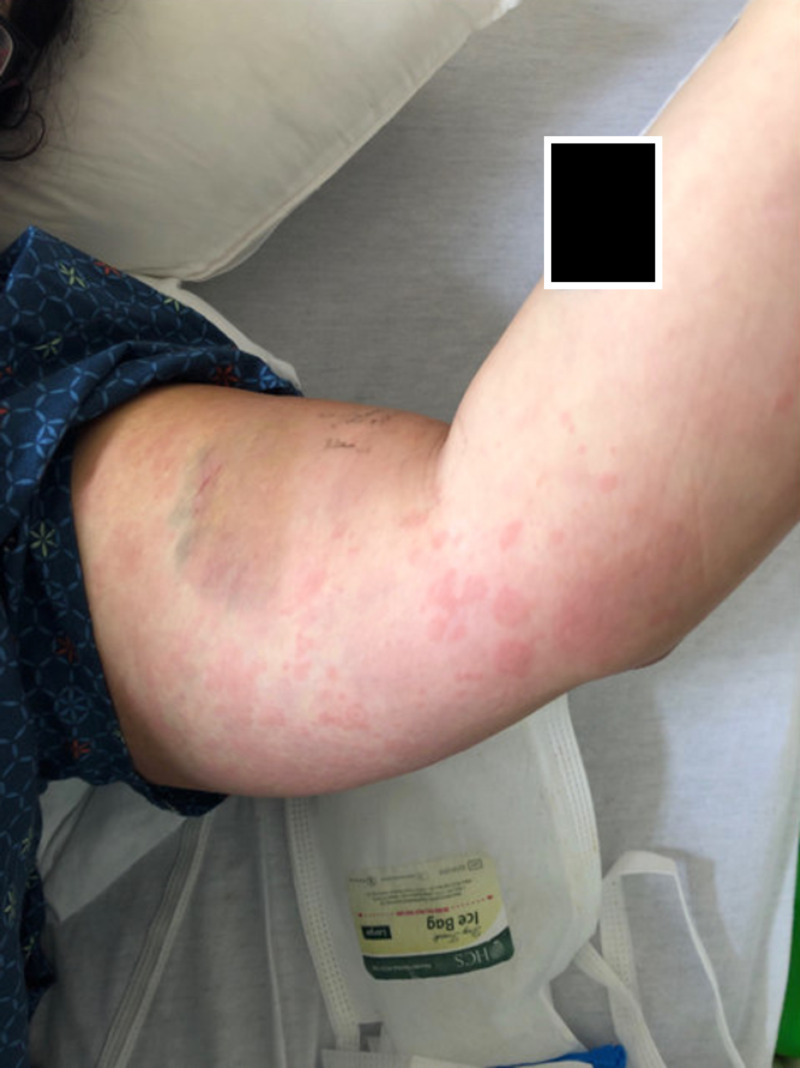
Maculopapular rash distributed over the upper extremity

She was seen for the same complaints in the emergency department. An X-ray of the bilateral knee was unremarkable; she was given morphine for pain and was sent for follow-up in the clinic. Meanwhile, suspecting a broad differential of autoimmune etiology, workup was done, including complete blood count, basic metabolic profile, liver function test, urine analysis, erythrocyte sedimentation rate (ESR), C-reactive protein (CRP), ANA, complements, rheumatoid factor, hepatitis panel, human acquired immunodeficiency virus (HIV) rapid antigen test, and blood cultures. She was admitted to the hospital for increasing pain and fever of 101 F. Her white blood cell (WBC) count at that time was 30.1, with a neutrophilic predominance and significant bandemia; the liver function test was abnormal. Bone marrow biopsy was not performed because of no lab or physical findings of any hematological malignancy. Suspecting infectious etiology she was started on broad-spectrum antibiotics, vancomycin, and cefepime. Her blood cultures and urine cultures were negative. Suspecting infectious endocarditis, echo was done, which was again negative for vegetations, with preserved ejection fraction and normal valves and wall motion. With the given amount of tachycardia, she was also having an elevation of D-dimer. Computed tomography (CT) chest with contrast was done, pulmonary embolism (PE) was ruled out, and the lungs were unremarkable for any pathology. She still had persisting spiking fevers every day and with each spike of fever, she had a worsening rash and joint pains. The rash was papulosquamous and present over extremities more than axial as shown in Figure 1 and Figure 2.

Infectious disease was involved, and investigations were done to rule out infectious as well as autoimmune causes. Chlamydia, gonorrhea, HIV, Hep B, Hep C, Epstein-Barr Virus (EBV), cytomegalovirus (CMV), Lyme’s serology, Borrelia Burgdorferi, and COVID-19 were negative. ESR and CRP were elevated with values of 98 and 298, respectively. Lactic acid was normal. ANA titers were 1:80 speckled pattern and the C2, C3, and C4 complements and C1Q levels were normal. Thyroid-stimulating hormone (TSH) was normal. The X-ray of the knee joints was unremarkable. She had a troponin elevation of 0.12, for which cardiac cath. was done, which showed clean coronaries. With all the infectious and autoimmune diseases ruled out, ferritin and transferrin were done, suspecting Still’s disease since she had temperature spikes, associated rashes, and mild troponin and ANA elevation, Ferritin was significantly elevated at 32,661 and transferrin was 115. With the clinical findings, four major criteria, and one minor Yamaguchi criteria along with the ferritin value, she was diagnosed to have adult-onset Still's disease and started on high-dose steroids when her rashes and joint pains decreased. She was given methylprednisolone 125 mg for three days and sent home with prednisone 60 mg. On follow-up after a week, she reported decreased symptoms. A plan was made to continue steroids for six to eight weeks and start on DMARDs after tapering the steroids.

## Discussion

Little is known about the etiological factors and pathophysiology of this rare autoimmune condition. Elevated levels of tumor necrosis factor-alpha (TNF-α), interleukin (IL)-6, and IL-18 are associated with ASOS [5]. ASOS has bimodal peaks: one in 15-25 years and the other one between 36 and 46 years, however, this patient was diagnosed at 30 years of age [6].

Patients with AOSD typically present with fever, rash, arthralgia, fever exceeding 102.2 F, and sore throat, as seen in this case, though sore throat was absent. The rash is typically nonpruritic and maculopapular and was seen distributed over the extremities in this case. The urticarial rash is a typical feature seen in ASOD, which was also present in this case. Apart from a sore throat, there was no lymphadenopathy, splenomegaly, or nervous system involvement, which might also be seen in case of ASOD [7].

The diagnosis of ASOD is based on Yamaguchi's criteria (Table 1), requiring five or more criteria, including two or more major criteria, yielding 96.2% sensitivity and 92.1% specificity and hyperferritinemia, along with the exclusion of other disorders [8].

**Table 1:** Yamaguchi criteria for Still's disease Abbreviations: IF, immunofluorescence; IgM, immunoglobulin M

Yamaguchi’s criteria
5 or more criteria are required, of whom 2 or more must be major.
Major criteria-
Fever >39 °C, lasting 1 week or longer
Arthralgia or arthritis, lasting 2 weeks or longer
Typical rash
Leukocytosis >10,000/mm3 with >80% polymorphonuclear cells
Minor criteria -
Sore throat
Recent development of significant lymphadenopathy
Hepatomegaly or splenomegaly
Abnormal liver function tests
Negative tests for antinuclear antibody (IF) and rheumatoid factor (IgM)

This case was diagnosed with four major criteria and one minor criterion, along with a ferritin level of 32,661.

ANA in this patient was positive, with a titer of 1/80 without any feature or other antibodies for SLE. It is very rare to have positive ANA with ASOD, and the diagnosis of ASOD should not be excluded based on the rheumatoid factor (RF) or ANA [9-10].

The treatment of AOSD includes NSAIDs. Patients who fail to respond to treatment should be started on DMARDs such as methotrexate. Biological agents are reserved for refractory cases, as rituximab was used in a case resulting in favorable outcomes [11].

## Conclusions

AOSD is a rare disease that is very challenging to diagnose. The lack of diagnostic tests and symptoms mimicking other diseases pose a major challenge for a physician in diagnosing AOSD. Through this case, we want to focus on the fact that ASOD should not be excluded in case of the presence of ANA and should be kept within the differentials in a patient presenting with fever, arthralgia, and rash.
